# Sleep Patterns of Pilots: An Objective Assessment

**DOI:** 10.7759/cureus.38983

**Published:** 2023-05-13

**Authors:** Mohammed Abdelaziz, Faris Alhejaili, Loai Alnouri, Abdulrahman Samman, Ahmed Alzehairi, Mansour Balkhyour, Ranya Alsumrani, Seithikurippu R Pandi-Perumal, Siraj O Wali

**Affiliations:** 1 ENT Department, Tameside Hospital, Ashton-under-Lyne, GBR; 2 Sleep Medicine Research Group, Sleep Medicine and Research Center, King Abdulaziz University Hospital, Jeddah, SAU; 3 Internal Medicine Department, Faculty of Medicine, King Abdulaziz University, Jeddah, SAU; 4 Faculty of Environmental Sciences, King Abdulaziz University, Jeddah, SAU; 5 Pulmonary Medicine, Somnogen Canada Inc, Toronto, CAN

**Keywords:** actigraphy, sleepiness, sleep, pilots, fatigue, aviation

## Abstract

Objectives

Circadian dysregulation (jetlag), sleep loss (extended wakefulness), sleep deprivation (acute or chronic), fatigue (exhaustion), preexisting medical and mental conditions, and medication use can all affect the cognitive and behavioral performance of commercial aircraft pilots. This study evaluated the sleep habits of pilots and copilots flying short-haul flights in the Gulf region.

Methods

This cross-sectional study examined Airbus A320 pilots and copilots for one of Saudi Arabia’s commercial airlines. Data such as age, sex, body mass index (BMI), employment position, work experience, flight hours, and rest time were collected. Each participant completed the Epworth Sleepiness Scale (ESS) for daytime sleepiness, the Pittsburgh Sleep Quality Index (PSQI), and the Fatigue Severity Index (FSI). Actigraphy equipment was used to perform objective sleep evaluations.

Results

Twenty-four participants were included. Actigraphy showed that 66.7% had an irregular sleep pattern and that 41.7% had poor sleep efficiency. We found that 12.5% had daytime sleepiness, 33% had poor sleep quality, and 29.2% had fatigue. We found a significant negative correlation between years of experience and time in bed; however, there was no significant difference in sleep time or sleep efficiency between pilots with different levels of experience.

Conclusions

We found that pilots and copilots are at risk of irregular sleep patterns, poor sleep efficiency, poor sleep quality, daytime sleepiness, and fatigue. This study emphasizes the need to instigate measures to minimize these risks.

## Introduction

The aviation industry has been growing rapidly in the Gulf region, particularly in the Kingdom of Saudi Arabia (KSA). The KSA currently has five airline companies, with substantial growth expected over the next decade. The expected growth in demand is anticipated to be greater than the necessary increase in personnel and expertise in the industry, resulting in an increased burden on the existing flight crew members [1].

Aviation is considered to be among the safest modes of transportation, with major accidents being rare (1.71 per million flights in 2020 (International Air Transport Association (IATA), 2021)). Unfortunately, a considerable proportion of these accidents have been linked to human error [2]. In assessments of various modes of transportation, longer working hours have been associated with a higher risk of adverse incidents, regardless of the mode of transportation [3-9]. This finding applies to aviation as well [10,11] and has been noted by the Subcommittee of Aerospace Human Factors, which has put forward measures to mitigate sleep disorders and fatigue among pilots [12].

In this study, we aimed to examine the sleep patterns of pilots and first officers (copilots) operating short-haul flights in the Gulf region. Although sleep disturbance and the risk of obstructive sleep apnea have been studied recently among pilots in the Gulf region [13,14], to our knowledge, no objective measurement has been used to accurately assess and measure sleep patterns and the possible implications of sleep disorders in this high-risk group. As such, this forms part of the focus of this study.

## Materials and methods

We conducted a cross-sectional study at the Sleep Medicine and Research Center (SMRC) of King Abdulaziz University Hospital (KAUH) on Airbus A320 pilots and copilots of one of the commercial airlines based in Saudi Arabia. Formal ethical approval (REF # 188-20) was obtained from the Ethical Committee at KAUH, Jeddah, Saudi Arabia.

Exclusion criteria included subjects still undergoing training, taking on management duties, or on leave for vacation or medical reasons. Participants with known sleep disorders or currently on medications for sleep-related disorders were excluded. Participants signed informed consent forms and were interviewed to obtain key data, including age, gender, body mass index (BMI), employment position, work experience, details of flying hours, and rest time. In addition, the Epworth Sleepiness Scale (ESS) for daytime sleepiness, the Pittsburgh Sleep Quality Index (PSQI), and the Fatigue Severity Index (FSI) were completed for each participant. Objective sleep assessment was performed by providing sleep diaries and actigraphy devices to each participant (Somnomedics GmbH- model number SOW106, Randersacker, Germany) to be used all the time for two continuous weeks according to the manufacturer’s instructions [15].

Subcommittee of aerospace human factors

Actigraphy

This is a validated method of objectively measuring sleep parameters and average motor activity over a specific duration using a noninvasive wrist-worn accelerometer [16]. Actigraphy is more accurate than self-reported sleep duration and more useful than sleep diaries in the assessment of patients with suspected sleep disorders. The main indication of actigraphy is the objective measurement of sleep-wake cycles, which can be used to complement self-reported sleep duration and other sleep parameters in patients with a range of sleep disorders, including insomnia, daytime sleepiness, circadian rhythm disorders and insufficient sleep syndrome. Actigraphy is relatively easy to use and does not require advanced operating procedures [17]. The sleep pattern is categorized into regular and irregular sleep patterns based on the visual assessment of the actigraphy results, indicating the individual's awake and sleep time in addition to the total hours of sleep per day. Furthermore, sleep efficiency (defined as the ratio of mean total sleep time to the duration of mean bedtime) was calculated using the estimated bedtime from the subjects’ sleep diaries and recorded sleep time from actigraphy. The normal sleep efficiency is considered equal to or more than 80%.

Epworth Sleepiness Scale (ESS)

Daytime sleepiness is subjectively assessed using the ESS. A score higher than 10 indicates excessive daytime sleepiness [18].

Pittsburgh Sleep Quality Index (PSQI)

The PSQI is used to assess the quality and pattern of sleep. Sleep is characterized as ‘poor’ or ‘good’ by evaluating seven domains: subjective sleep quality, sleep latency, sleep duration, habitual sleep efficiency, sleep disturbances, use of sleep medication, and daytime dysfunction over the last month. Scoring of the answers is based on a 0 to 3 scale, with a higher score indicating lower quality of sleep in that particular domain. An overall score higher than 5 indicates poor sleep [19].

Fatigue Severity Index (FSI)

The FSI is a validated tool used to assess fatigue among patients with a variety of medical and neurologic disorders. It consists of nine items, each scored from one (strongly disagree) to seven (strongly agree), with total scores ranging from nine to 63. The higher the score, the more profound the fatigue and its perceived effect on a person’s daily activities [20].

Statistical analysis

Statistical measures were derived using the IBM SPSS Statistics for Windows, Version 22.0 (Released 2013; IBM Corp., Armonk, New York, United States). The categorical variables are summarized and presented using tables. Measures used to analyze numerical variables included the mean, standard deviation, Spearman’s correlation, chi-square test, and Mann-Whitney U test. The results were considered significant if p values were less than 0.05, with 95% confidence intervals (CIs).

## Results

Demographics

Data were collected for 39 participants. Fifteen participants were excluded based on the exclusion criteria; thus, 24 participants were included: 10 pilots (41.7%) and 14 first officers (58.3%). The mean age and BMI were 41 years (SD = 7.4) and 25.6 kg/m2 (SD = 3.8), respectively. Only 8% of participants had more than 15 years of experience; however, the majority of participants (58.3%) performed ≥40 takeoffs (T/O) and landings per month. Furthermore, 12.5% performed night flights exclusively, and 79.2% performed a mix of morning and night flights (Table 1).

**Table 1:** Demographic data of participants BMI: body mass index

	n	%
Rank		
Captain	10	41.7%
First Officer	14	58.3%
Experience (years)		
≤15	22	91.7%
>15	2	8.3%
Range	6-19	
Mean ± SD	11.333±3.655	
Age (years)		
Less than 30	1	4.2%
30-39	12	50.0%
40-49	9	37.5%
50+	2	8.3%
Range	26-56	
Mean ± SD	41.0 ± 7.407	
Number of takeoffs & landings/month		
≤35	6	25%
36-45	15	62.5%
>45	3	12.5%
Range	29-45	
Mean ± SD	39.01 ± 5.099	
Typical daily duty time		
Less than 8 hours	1	4.2%
8-14 hours	22	91.7%
More than 14 hours	1	4.2%
Timing of flights		
Daytime	2	8.3%
Nighttime	3	12.5%
Both	19	79.2%
BMI		
< 30	21	87.5%
> 30	3	12.5%
Range	20.02-38.06	
Mean ± SD	25.64 ± 3.83	

Sleep quality/quantity measures

We found that healthy pilots and copilots were at risk of poor sleep quality and irregular sleep patterns. Objective assessment using actigraphy for 24 participants showed that 66.7% had an irregular sleep pattern, and 41.7% of all participants had poor sleep efficiency. Subjective assessment of all participants using the validated questionnaires revealed that 12.5% had daytime sleepiness based on the ESS, 33% had poor sleep quality based on the PSQI, and 29.2% had fatigue based on the FSI (Table 2).

**Table 2:** Sleep pattern, sleep quality, fatigue and sleepiness among the participants. ESS: Epworth Sleepiness Scale; PSQI: Pittsburgh Sleep Quality Index; FSI: Fatigue Severity Index

Measure		Data		Score	
		n	%	Range	Mean ± SD
ESS (Sleepiness)	No	21	87.5%	0-12	5.542 ± 3.526
	Yes	3	12.5%		
PSQI (Sleep Quality)	Good	16	66.7%	0-9	3.500 ± 2.414
	Poor	8	33.3%		
FSI (Fatigue)	No	17	70.8%	9-45	26.583 ± 12.297
	Yes	7	29.2%		
Actigraphy-based sleep pattern	Regular sleep	8	33.3%	-	-
	Irregular sleep	16	66.7%		
Actigraphy-based sleep efficiency	Normal	14	58.3%	34.5-97	75.380 ± 18.763
	Abnormal	10	41.7%		

However, the correlation between demographic data and the sleep variables, i.e., excessive sleepiness, fatigue, poor sleep quality, sleep efficiency, sleep duration and time in bed, showed a significant moderate negative correlation between years of experience and time in bed. Similarly, there was a significant negative correlation between the number of T/O and landings per month and sleepiness (Table 3).

**Table 3:** Correlations between demographic data (age, experience, number of T/O and landings/month, typical daily duty time, and BMI) and ESS, PSQI and FSI scores, time in bed, total sleep time and sleep efficiency ESS: Epworth Sleepiness Scale; PSQI: Pittsburgh Sleep Quality Index; FSI: Fatigue Severity Index; BMI: body mass index; T/O: takeoffs

		Age	Experience	Number of T/O & landings/month	Typical daily duty time	BMI
ESS	r	-0.327	-0.279	-0.423	-0.220	-0.126
	p value	0.119	0.186	0.039	0.302	0.559
PSQI	r	-0.168	0.068	-0.108	-0.328	-0.170
	p value	0.432	0.753	0.615	0.118	0.428
FSI	r	0.024	0.021	0.322	-0.115	0.218
	p value	0.911	0.922	0.126	0.593	0.305
Time in bed	r	-0.294	-0.524	-0.220	-0.282	0.109
	p value	0.164	0.009	0.303	0.182	0.611
Total sleep time	r	-0.038	-0.171	0.126	-0.063	-0.042
	p value	0.859	0.424	0.556	0.772	0.846
Sleep efficiency	r	0.079	0.349	-0.239	-0.178	-0.008
	p value	0.742	0.132	0.311	0.452	0.972

## Discussion

This study demonstrated that healthy pilots and copilots are at risk of poor sleep quality and irregular sleep patterns. The objective actigraphic assessment of 24 participants showed that 66.7% had an irregular sleep pattern, and 41.7% of all participants had poor sleep efficiency. Furthermore, one-third of participants were found to have poor sleep quality. The frequencies of sleepiness and fatigue in this sample were 12.5% and 29.2%, respectively. Although there was no correlation between the different demographic parameters and sleep disturbances, there was a moderate negative correlation between years of experience and time in bed (r= -0.524; p=0.009), yet there seemed to be no significant difference in sleep time and sleep efficiency for the more experienced participants compared to the others. Surprisingly, our study also showed a significant negative correlation between the number of T/O and landings per month and sleepiness as measured by the ESS.

Due to the high risks associated with flying, it is understandably essential for airline pilots and copilots to remain alert to ensure a high standard of flight safety. Our study revealed that airline pilots and copilots have a high prevalence of poor sleep quality and fatigue. Gregory et al. [21] reported that over 84% of American air medical pilots had fatigue that compromised their flight performance, with just under 28% reporting ‘nodding off’ during flight. Many studies have reported factors that lead to fatigue, including long daily working hours, night work, and reduced resting time between working days [22-24].

More recently, Marqueze et al. [24] conducted a cross-sectional study of 1,235 Brazilian airline pilots who work on national or international flights and found the prevalence of sleepiness while flying the airplane to be as high as 57.8%. This was found to be related to flying for more than 65 hours a month, frequent technical delays, greater need for recovery after work, below-optimal work ability, and insufficient sleep. While our study showed a lower rate of sleepiness in our population at 12.5% (ESS), the objective actigraphic assessment revealed that 66.7% of participants had an irregular sleep pattern and 41.7% had poor sleep efficiency. Understandably, the demanding nature of the job likely contributes to these findings. Conversely, our study demonstrated a significant negative correlation between the number of T/O and landings per month and hypersomnolence, which may be difficult to explain. Interestingly, Zakariassen and Bjorvatn examined the causes and strategies for combating sleepiness among pilots working in two different helicopter emergency medical services operating with different shift systems [25]. They compared Norwegian Air Ambulance pilots, who performed helicopter missions 24/7 when on duty, with Austrian Air Ambulance pilots, who performed missions that started in daylight. These authors found no significant difference in sleepiness as measured by the ESS between the two groups, despite a different pattern of work. Moreover, these authors found that the Norwegian pilots kept themselves busy as a sleepiness management strategy, indicating that activity may be a way of counteracting sleepiness [25]. Eriksen et al. [26] also found that activity may be a way to combat sleepiness. These authors examined sleepiness throughout the day at hourly intervals and during controlled activities such as reading, writing, walking and social interaction and found that walking and social interaction were associated with low sleepiness compared to sedentary and quiet office work [26].

As this study and others demonstrated that airline staff suffer from interrupted sleep leading to fatigue and sleepiness, it is not surprising that the U.S.-based Flight Safety Foundation has recommended countermeasures, including fatigue risk management policies, crew reporting mechanisms, mandatory in-flight rest periods, scheduled onboard bunk sleep periods, and strategies for addressing any flight disruptions or changes in schedules [12]. This study highlights the importance of instigating these measures to minimize the risk of adverse flight incidents.

Our study may have a few limitations. Although hypersomnolence may be diagnosed by a subjective questionnaire such as the ESS, subjective measures, such as negative symptoms experienced by participants, may be at risk of being underreported due to the negative connotations and perceived risks to career progression associated with providing negative answers. Although we used an objective method for the assessment of the sleeping patterns with actigraphy, more objective tests such as the Multiple Sleep Latency Test (MSLT) and the Maintenance of Wakefulness Test (MWT) may be of benefit to confirm these diagnoses and remove or mitigate these reporting biases. Unfortunately, neither of these two tests was performed in our study; however, they may be considered in future follow-up studies when addressing sleepiness among airline personnel. Other limitations include the small sample size, which limits the conclusions that can be drawn from statistical analyses, especially the correlations noted between demographic measures and sleep measures.

## Conclusions

In summary, our study showed that pilots and copilots are at risk of irregular sleep patterns, poor sleep efficiency, poor sleep quality, daytime sleepiness, and fatigue. In particular, our study demonstrated that two-thirds of the participants objectively had irregular sleep and that 41.7% had poor sleep efficiency. This trend towards poor sleep patterns in pilots and copilots needs further studies with a larger number of participants to evaluate the degree and magnitude of the problem. More studies are needed to examine the work-related factors that contribute to sleep disorders in this group and to address measures to reduce the associated risks.
